# Natural history of uncomplicated urinary tract infection without antibiotics: a systematic review

**DOI:** 10.3399/bjgp20X712781

**Published:** 2020-09-22

**Authors:** Tammy Hoffmann, Ruwani Peiris, Chris Del Mar, Gina Cleo, Paul Glasziou

**Affiliations:** Institute for Evidence-Based Healthcare, Faculty of Health Sciences and Medicine, Bond University, Gold Coast.; Institute for Evidence-Based Healthcare, Faculty of Health Sciences and Medicine, Bond University, Gold Coast.; Institute for Evidence-Based Healthcare, Faculty of Health Sciences and Medicine, Bond University, Gold Coast.; Institute for Evidence-Based Healthcare, Faculty of Health Sciences and Medicine, Bond University, Gold Coast.; Institute for Evidence-Based Healthcare, Faculty of Health Sciences and Medicine, Bond University, Gold Coast.

**Keywords:** cystitis, general practice, natural history, primary care, urinary tract infections

## Abstract

**Background:**

Although uncomplicated urinary tract infection (UTI) is commonly treated with antibiotics, the duration of symptoms without their use is not established; this hampers informed decision making about antibiotic use.

**Aim:**

To determine the natural history of uncomplicated UTI in adults.

**Design and setting:**

Systematic review.

**Method:**

PubMed was searched for articles published until November 2019, along with reference lists of articles identified in the search. Eligible studies were those involving adults with UTIs in either the placebo group of randomised trials or in single-group prognostic studies that did not use antibiotics and measured symptom duration. A modified version of a risk of bias assessment for prognostic studies was used. Outcomes were the percentage of patients who, at any time point, were symptom free, had symptom improvement, or had worsening symptoms (failed to improve). Adverse event data were also extracted.

**Results:**

Three randomised trials (346 placebo group participants) were identified, all of which specified women only in their inclusion criteria. The risk of bias was generally low, but incomplete reporting of some details limited assessment. Over the first 9 days, the percentage of participants who were symptom free or reported improved symptoms was reported as rising to 42%. At 6 weeks, the percentage of such participants was 36%; up to 39% of participants failed to improve by 6 weeks. The rate of adverse effects was low and, in two trials, progression to pyelonephritis was reported in one placebo participant.

**Conclusion:**

Although some uncertainty around the natural history of uncomplicated UTIs remains, some women appear to improve or become symptom free spontaneously, and most improvement occurs in the first 9 days. Other women either failed to improve or became worse over a variable timespan, although the rate of serious complications was low.

## INTRODUCTION

Uncomplicated urinary tract infections (UTIs) are very common in general practice, and UTI symptoms can be burdensome and impact a person’s quality of life.^1^ Antibiotics are usually used to manage UTIs on the assumption that they reduce the duration and severity of symptoms and complications. However, evidence from randomised trials about the efficacy of antibiotics in UTIs shows that the benefit/harm trade-off of antibiotic use must be considered.^2^ Additionally, it is known that, as well as possible harms to individual patients, antibiotic use promotes antibiotic resistance — a serious threat to modern medicine.^3^

Clinicians and patients overestimate the benefits and underestimate the harms of treatments;^4^ for acute respiratory infections, for example, they greatly overestimate the reduction in symptom duration from antibiotic use.^5^ As well as inaccurate expectations about antibiotic benefits, clinicians and patients may have poor awareness about possible antibiotic harms, including resistance, and the natural course of infections. Without such information, evidence-based decision making — in which the benefits and harms of antibiotic use are carefully considered,^4^ along with patients’ preferences and values — is not possible.

As far as the authors are aware, there is no known synthesis of the research regarding the resolution of symptoms and complications if antibiotics are not used in the management of uncomplicated UTIs in adults; as such, this review aimed to determine the natural course of symptoms of such patients.

## METHOD

### Eligibility criteria

The reviewers aimed to identify studies that met the following criteria:

- included adults who had uncomplicated UTI;
- reported outcome data on the duration of UTI symptoms; and
- included a group that received no therapeutic treatment (that is, placebo or ‘no treatment’) — this could be either a comparison group in a randomised trial or a single-group prognosis study (such as a cohort study).

Studies were excluded if the study population had any of the following features:

- asymptomatic UTIs;
- asymptomatic bacteriuria;
- complicated UTI (for example, pyelonephritis and sepsis);
- recurrent UTI;
- chronic UTI;
- emphysematous cystitis;
- *Candida* infection;
- haemorrhagic cystitis;
- interstitial cystitis; or
- schistosomiasis.

**Table:** How this fits in

Uncomplicated urinary tract infections (UTIs) are a very common reason for general practice consultations and one of the most common reasons for the prescription of antibiotics. Informed decision making should consider the benefit/harm trade-off of antibiotic use and the natural course of the illness. The studies reviewed here, which focused solely on women, demonstrated that UTI symptoms resolve spontaneously in approximately a third of women in the first 7–10 days. Current guideline recommendations from the National Institute for Health and Care Excellence are to delay prescribing by 2 days but the findings of this systematic review indicate that this may be too short a timeframe.

### Search methods

In May 2019, a search was conducted using PubMed to identify systematic reviews (as a method of identifying potentially eligible randomised trials) and prognosis studies (search strings are detailed in Supplementary Box S1). Potentially relevant articles were also identified from the references of articles identified during the screening process. An updated search was conducted on 20 November 2019, but no further eligible articles were found.

### Screening and eligibility assessment

One researcher screened the search results and held discussions with a second researcher about eligibility, as needed. Articles in foreign languages were potentially eligible and translated for screening purposes using Google Translate.

### Risk of bias assessment and data extraction

As the reviewers were focused on using prognosis outcome data, a modified version of a risk of bias assessment framework proposed by Altman *et al* ^6^ was used to assess the included studies. The following study and methodological quality characteristics were also extracted from each included study:

- country;
- study design;
- sample size;
- participant age;
- participant sex;
- UTI diagnosis criteria;
- method of randomisation;
- exclusion criteria;
- concurrent treatments; and
- duration of follow-up.

To obtain information regarding the proportion of patients who were symptom free, data were extracted from the studies, either directly from the published text and tables or, where needed, by using WebPlotDigitizer extraction software (https://automeris.io/WebPlotDigitizer) to retrieve values from the figures. Where possible, in those trials in which participants left the placebo group because of worsening symptoms and commenced antibiotics, the percentage outcome data were calculated using as the denominator the total number of participants who were initially randomised to the placebo group.

### Outcomes

Data were extracted for the following outcomes from participants in the placebo group:

- ‘symptom free’ — percentage of participants who were symptom free at any time point;
- ‘symptoms improved’ — percentage of participants who had improved symptoms at any time point; and
- ‘failure to improve’ — percentage of participants who had worsening of symptoms at any time point.

Data were also extracted regarding the rate of crossover from placebo to antibiotic groups, reasons for the crossover, and adverse events in both treatment and placebo group participants. In some studies, percentage values were given for a time range (for example, ‘x’% symptom free at 5–7 weeks); in these cases, the median time point was used to present values graphically (for example, ‘x’% symptom free at 6 weeks).

### Data analysis

Outcome data were graphed on scatter plots against time to enable visualisation of the rate of symptom resolution. The diameter of the data points on the scatter plots was adjusted to represent each study’s sample size.

## RESULTS

**Figure 1.**
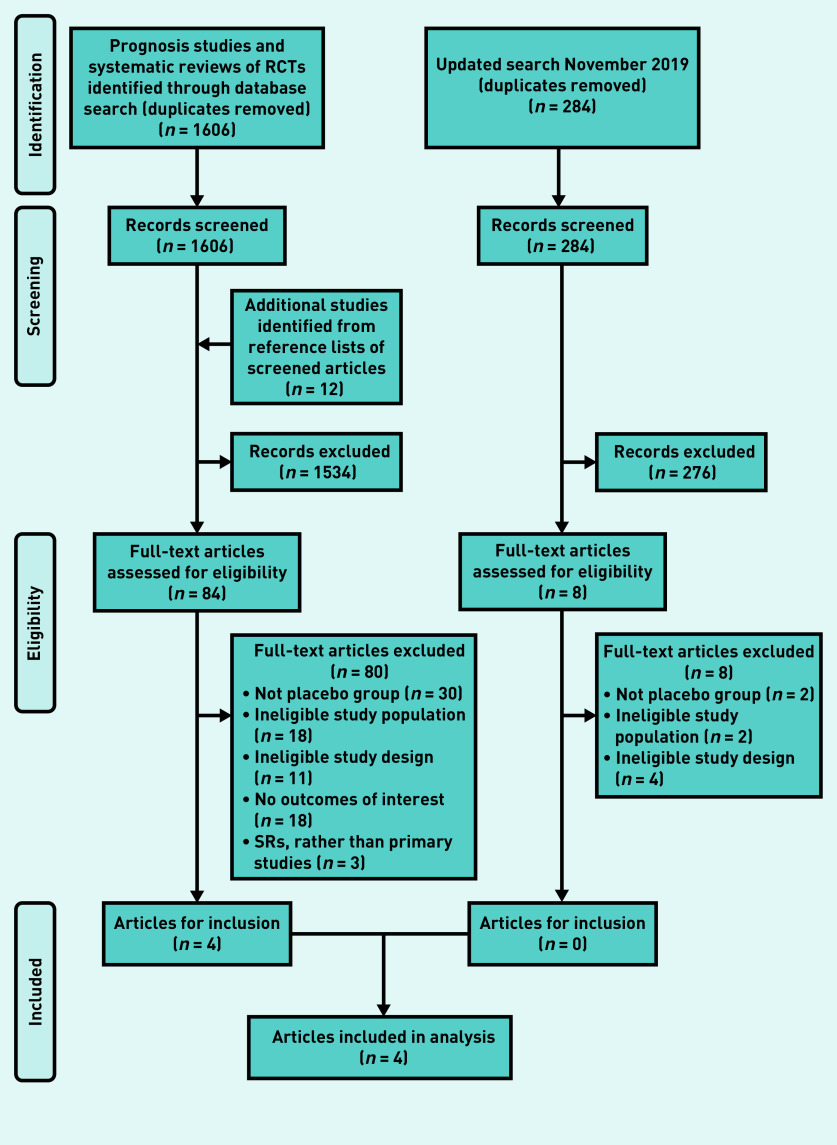
***Flowchart of study recruitment. RCT = randomised controlled trial. SR = systematic review.***

The article selection process is outlined in Figure 1. Four articles^7^^–^^10^ met the eligibility criteria and were included. Two of the included articles were from one trial conducted by Ferry *et al*;^9^^,^^10^ these articles use data taken from the same cohort of patients and, as such, were treated as one dataset. The 2007 article^10^ reported outcomes from a bacterial culture-positive subset of participants. Data on symptom duration were reported in, and extracted from, the 2004 article;^9^ adverse effects were reported in, and extracted from, the 2007 article.^10^

Table 1 details characteristics of the included studies. There were 346 placebo participants in total, with Ferry *et al* ’s trial providing the majority (83%) of these. All participants were non-pregnant adult women, as this was an inclusion criterion for all three trials. Mean age was not consistently reported and the age criteria across the studies ranged from 15–82 years. There was variation in the diagnostic criteria, with dysuria the only criterion common to all three studies. Follow-up was up to 3 months in one study,^7^ but, for the outcomes relevant to this review, the longest follow-up time was 6 weeks. Most of the outcome data were collected within 2 weeks.

**Table 1.** Characteristics of included studies

**Characteristic**	**Study**		
	**Brooks *et al* (1972)^7^**	**Christiaens *et al* (2002)^8^**	**Ferry *et al* (2004;^9^ 2007^10^)**
**Country**	England	Belgium	Sweden
**Study design**	Randomised controlled trial	Randomised controlled trial	Randomised controlled trial
**Placebo group participants, *n***	20	38	288
**Age criteria, years**	15–75	15–54	18–82
**Sex**	All female	All female	All female
**Diagnosis inclusion criteria**	Dysuria Frequency (a subset of participants with urine<10^4^organisms/ml was randomised)	Dysuria Frequency Urgency Pyuria	Dysuria Urgency Suprapubic pain Loin pain Minimum severity score ≥2
**Exclusion criteria**	Antibiotic allergy Pregnancy Concurrent antibiotic use Cardiac, renal, or hepatic failure	Antibiotic allergy Pregnancy Recent antibiotic use Diabetes Fever Gynaecological symptoms Immunocompromised Recurrent UTI Renal structural abnormalities	Antibiotic allergy Pregnancy Recent antibiotic use Genital infection Pyelonephritis Urinary incontinence
**Randomisation method**	Randomisation code used, provided by pharmaceutical company	Randomisation list used, no further details	Randomisation stated, but no further details
**Maximum follow-up period^a^**	11 days	7 days	6 weeks

a Duration of follow-up for the outcomes included in this study only. UTI = urinary tract infection.

Randomisation was reported in all studies, although little detail of the randomisation method was provided (Table 2). In one study,^7^ two trials were conducted from one recruitment process: the trial eligible for this review involved a subset of participants classified as bacteriuria negative (urine <10^4^ organisms/ml) who were randomised to either placebo or an antibiotic; the other trial was in women with a significant bacteriuria count (that is, ≥10^5^ organisms/ml) and compared two antibiotics. Using the selected elements of Altman’s risk assessment framework,^6^ a low-tomoderate risk of bias was determined for the prognostic outcome data.

**Table 2.** Risk of bias assessment

**Category**	**Criteria**	**Study**		
		**Brooks *et al* (1972)^7^**	**Christiaens *et al* (2002)^8^**	**Ferry *et al* (2004;^9^ 2007^10^)**
Defined sample	Description of source of patients, and inclusion and exclusion criteria	Yes	Yes	Yes
Representative sample	Participants selected as consecutive cases	Yes	Not reported	Not reported
Follow-up rate	Outcome data available for at least 80% of participants at one follow-up point	Yes	Yes	57% at 5–7 weeks, but>80% at 8–10 days
Prognosis	Raw data, percentages, survival rates, or continuous outcomes reported	Yes	Yes	Yes

### Synthesis of results

#### Interpretation of time points

The time points for the data values were not clearly defined in the studies. In Ferry *et al* ’s trial, symptoms were measured from inclusion or the first day of treatment, but the number of days patients had symptoms prior to this was not reported. Those trials by Brooks *et al* and Christiaens *et al* defined neither the starting point for the time points of measured symptoms nor the duration of symptoms prior to study inclusion.

#### Symptom free or symptom improvement

**Figure 2.**
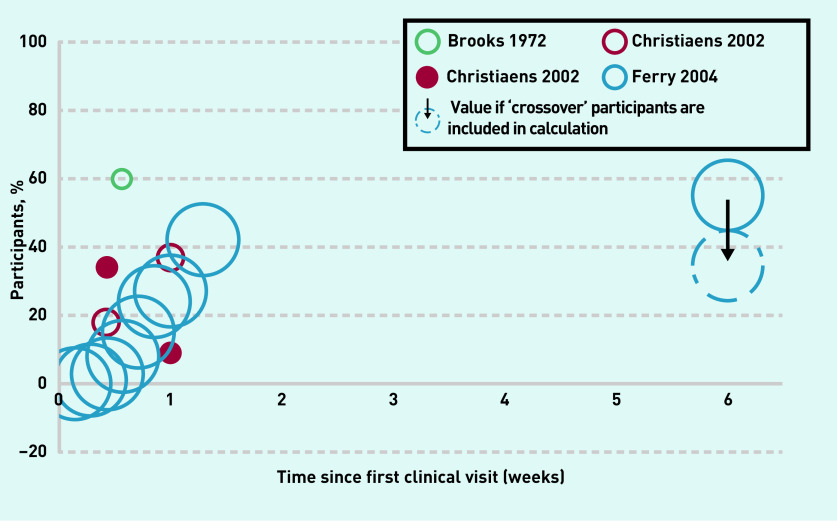
***Symptom progression. Participants reporting being ‘symptom free’ (outlined circle) or having ‘symptom improvement’ (solid circle).^a,b^*** ***^a^ Diameter of circles represents weighting by study sample size. Crossover refers to participants in the placebo group who had worsening symptoms and had commenced antibiotic treatment by the time of follow-up.*** ***^b^The first 7 days of data from the Ferry 2004 study^9^*** ***come from the participant daily symptom diary and are not shown inTable 3***.

Symptom improvement over the first 9 days shows a rise, to a maximum of 42% of participants (Figure 2, Table 3). At 6 weeks there was only one data point at 54%, or 36% of participants after the authors adjusted for crossovers (Table 3).

**Table 3.** Symptom progression results

**Outcome**	**Time point^a^**	**Placebo group, *n*/*N* (%)**	**Definition given in trial**
**Symptom free**			
Christiaens *et al* (2002)^8^	3 days	7/38 (18)	No symptoms
Brooks *et al* (1972)^7^	4 days	12/20 (60)	Clear of symptoms
Christiaens *et al* (2002)^8^	7 days	14/38 (37)	No symptoms
Ferry *et al* (2004)^9^	7 days	78/277 (28)	No symptoms
Ferry *et al* (2004)^9^	9 days	(42)^b^	No symptoms
Ferry *et al* (2004)^9^	6 weeks	90/166 (54→36)^c^	No symptoms
**Symptom improvement**			
Christiaens *et al* (2002)^8^	3 days	12/35 (34)	A few symptoms
Christiaens *et al* (2002)^8^	7 days	3/33 (9)	A few symptoms

**Failure to improve**			
Christiaens *et al* (2002)^8^	2 days	6/38 (16)	5 with worsening symptoms; 1 with suspected acute pyelonephritis (‘crossover’)
Christiaens *et al* (2002)^8^	5 days	6+5^c^ = 11/38 (29)	Worsening symptoms, commenced antibiotics (‘crossover’)
Brooks *et al* (1972)^7^	7 days	5/20 (25)	Still had symptoms
Ferry *et al* (2004)^9^	6 weeks	(39)	Worsening symptoms, commenced antibiotics (‘crossover’)

a *Time points are from commencement of placebo treatment for Ferry* et al*’s (2004)^9^study; for Brooks* et al *(1972)^7^and Christiaens* et al *(2002); ^8^the context for the time points is not specified. In all studies, the relationship between recorded time points and the onset of symptoms is unknown. If time points were reported as a range in the original study, the median was taken and used for analysis.*

b *Number not given in trial; extracted from Ferry* et al’*s (2004)^9^ data; see Figure 3*.

c Reported as 54%, but if ‘crossover’ participants are included, this reduces to 36%, as per the authors’ adjustment for dropouts. ^c^6 at Day 2, and then 5 additional patients at 5 days = 11 who had ‘failed to improve’ by Day 5.

#### Individual symptoms

Data for individual symptoms were only available from Ferry *et al* ’s (2004) article and resolution in 100% of participants was not seen for any symptom, even at 6 weeks. Throughout the follow-up period, a larger percentage of participants were free of suprapubic pain and loin pain compared with dysuria and urgency, for example, at 9 days, 82% of participants were free of suprapubic pain and 85% free of loin pain, compared with 65% for dysuria and 62% for urgency (Figure 3).

**Figure 3.**
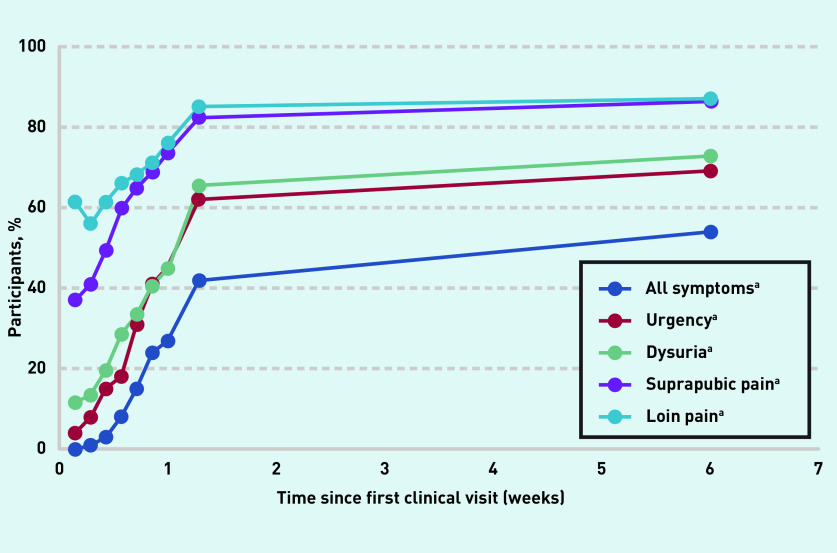
***Symptom progression. Participants reporting being symptom free, by individual symptoms and all symptoms.*** ***^a^Results all from Ferry SA, Holm SE, Stenlund H, et al . The natural course of uncomplicated lower urinary tract infection in women illustrated by a randomized placebo controlled study. Scand J Infect Dis 2004.****^9^* ***Reprinted by permission of Informa UK Limited, trading as Taylor & Francis Group, www.tandfonline.com.***

#### Failure to improve, and crossover to antibiotic treatment

**Figure 4.**
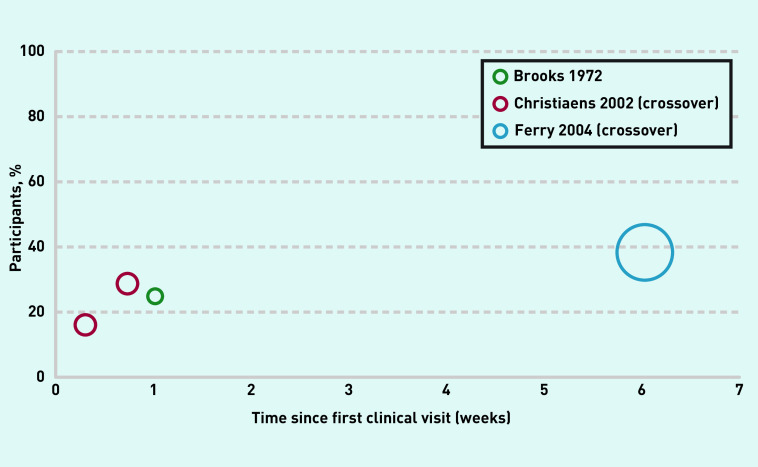
***Symptom progression. Participants with clinical failure to improve.^a^*** ***^a^Diameter of circles represents weighting by study sample size. Crossover refers to participants in the placebo group who had worsening symptoms and had commenced antibiotic treatment by the time of follow-up.***

The percentage of participants who failed to improve over 6 weeks ranged from 16% to 39%, with a large proportion of participants in the placebo group in Ferry *et al* ’s trial commencing antibiotics because of worsening symptoms (Figure 4, Table 3). Only Brooks *et al* ’s study did not measure failure to improve explicitly, but rather recorded the number of participants with persisting symptoms. In the studies by Ferry *et al* and Christiaens *et al*, rates of leaving the trial because of persistent or worsening symptoms were also interpreted as ‘failure to improve’ and, as such, were included in the review analysis (Figure 4, Table 3). Christiaens *et al* reported numbers and reasons for placebo participants commencing antibiotics.

In Ferry *et al* ’s trial, a large proportion of the placebo cohort dropped out prior to the 6-week follow up, because of non-resolving or worsening symptoms, and commenced antibiotics; these participants were also considered as having failed to improve. These ‘crossover’ numbers are not represented in the symptom-free data reported by the authors in the articles. The authors reported that, if these crossovers were accounted for at 6 weeks, the percentage of patients who were symptom free would be 36% rather than 54% (Figure 2). The crossover numbers are not reported for the other time points in Ferry *et al* ’s study — it is likely that the true values at these time points would also be lower. The reviewers were unable to reconcile a discrepancy in the data in Ferry *et al* ’s 2004 article: the value of 42% at 9 days is from extracted data derived from a figure included in the article, whereas the reported value in a table in the same article suggested the value was 28%.

#### Adverse effects and serious complications

The incidence of generalised adverse effects in the placebo groups did not appear to be markedly higher than those in the treatment groups (Table 4). The progression to pyelonephritis or other serious complications was uncommon (Table 4).

**Table 4.** Incidence rates of adverse events and serious complications^a^

**Study**	**Time point**	**Placebo group, *n*/*N* (%)**	**Adverse event/serious complication**	**Treatment group, *n*/*N* (%)**	**Adverse event/serious complication**
**Adverse effects**					
Christiaens *et al* (2002)^8^	2 days	10/38 (26)	Gastrointestinal problems (*n* = 3), headache (*n* = 3), dizziness/fatigue (*n* = 3), sleep disturbances (*n* = 0), vaginal itching (*n* = 2), dermatological problems (*n* = 1), other (*n* = 2)	9/40 (23)	Gastrointestinal problems (*n* = 4), headache (*n* = 2), dizziness/fatigue (*n* = 2), sleep disturbances (*n* = 1), vaginal itching (*n* = 1), dermatological problems (*n* = 0), other (*n* = 0)
Brooks *et al* (1972)^7^	7 days	1/20 (5)	General malaise (*n* = 1)	7/24 (29)	General malaise (*n* = 1), sore mouth (*n* = 1), sore tongue (*n* = 2), black, furred tongue (*n* = 1), allergic skin rash (*n* = 1), vomiting (*n* = 1)
Ferry *et al* (2007)^10^	6 weeks	(4)	Gastrointestinal reaction	(5–8)	Gastrointestinal reaction

**Serious complications**					
Christiaens *et al* (2002)^8^	2 days	1/38 (3)	Pyelonephritis	None reported	None reported
Ferry *et al* (2007)^10^	6 weeks	1/20 (5)	Pyelonephritis	1/24 (4)	Pyelonephritis
Brooks *et al* (1972)^7^		None reported	None reported	None reported	None reported

a *Some patients experienced* >*1 adverse event.*

## DISCUSSION

### Summary

This review of studies that reported on the natural course of UTI symptoms in adults who were managed without antibiotic treatment identified placebo groups from three randomised trials. All three studies included women only; one trial^9^ predominated with the most data and the largest number of participants. The reviewers found that some women appear to improve or become symptom free without the use of antibiotics, with most improvement in the first 9 days. However, some women who did not receive antibiotic treatment either failed to improve or became worse, over a variable timespan, although the rates of serious complications (for example, pyelonephritis) were low.

### Strengths and limitations

This review adopted a novel approach to providing prognostic information that is important for clinical decision making regarding this condition by examining the natural history data that were available from placebo-controlled trials. A possible limitation is that systematic reviews were searched for as a method of identifying relevant randomised trials, as new trials may have been published since the most recently published systematic review conducted its search. However, this pragmatic approach was taken as a number of systematic reviews of trials involving the use of antibiotics for UTIs have been conducted and would have already identified potentially eligible trials. Heterogeneity (such as in UTI definition, symptom definition, and timing of outcome measurement) precluded a meta-analysis being conducted.

Other limitations with the available data include:

- unclear duration of symptoms prior to study inclusion;
- loss of follow-up data from some participants who left the placebo groups to commence antibiotics;
- most of the findings derived from one study;
- variation in the studies’ inclusion and exclusion criteria — for example, Brooks *et al* ’s study comprised women with symptoms who had negative or non-significant bacteriuria; and
- unclear reporting on details of randomisation methods.

The unclear reporting of randomisation methods limited the ability to identify biases, but, in the two trials that reported baseline characteristics, these were similar; this suggests the randomisation was adequate.

In the three trials included in the review, outcome definitions differed, for example, ‘improvement’ (symptom severity versus number of symptoms) and ‘relapse/failure’ (defined by bacteriuria versus symptoms versus both). No studies reported on the use of over-the-counter medications — although their use may influence symptom scores, this is also reflective of what many women with UTI are likely to do.^11^ Most treatments for symptoms have not been evaluated in placebo-controlled trials (for example, urinary alkalisers^12^), which also precludes their inclusion in this review as an additional source of natural history data.

### Comparison with existing literature

The body of evidence to inform the natural history of uncomplicated UTI does not appear to be expanding. A systematic review and meta-analysis published in 2009^2^ on the effectiveness of antibiotics for UTIs identified the three placebo-controlled trials included in this current review, as well as another two that did not meet the inclusion criteria because it was unclear whether, and how, they measured symptom resolution.^13^^,^^14^ An observational study, conducted between 2002 and 2005, of women with suspected uncomplicated UTI reported a mean symptom duration of 3.83 days in the 511 women who saw a clinician for their symptoms and rated the problem as moderately bad or worse;^15^ however, in that sample, only 17 participants (approximately 3%) did not take antibiotics and their mean symptom duration was 4.94 days.

It was noted that loin pain, usually associated with renal involvement of a UTI, appeared to be present at study inclusion in 40% of participants in Ferry *et al* ’s trial. This seems unusually high; Ferry *et al* (2004)^9^ suggested the symptom diary method of data collection used may have contributed to this and, therefore, documented an incidence rate that was, likely, more accurate but higher than those reported in other studies.

### Implications for practice and research

There are insufficient data to be certain about the natural history, including the duration of symptoms and rate of recovery, of uncomplicated UTI that is not managed with antibiotics. The available evidence shows that some UTIs resolve spontaneously over a few weeks. The reviewers found that, at 6 weeks, up to a third of women, approximately, who did not receive antibiotics were symptom free, with approximately another third requiring antibiotics for worsening symptoms between 1 and 6 weeks.

There is inconsistency and a lack of evidence in guideline recommendations about the use of antibiotics for uncomplicated UTI. As an example, guidelines from the Infectious Disease Society of America do not mention using a delayed prescribing approach and consider withholding antibiotics as unjustified,^16^ whereas guidelines for the UK published by the National Institute for Health and Care Excellence (NICE) recommend a delayed prescribing approach by commencing antibiotic use if there is no symptom improvement in 2 days.^17^ Therapeutic Guidelines (Australia) state that most women aged <65 years become symptom free within 1 week without the use of antibiotics.^18^ The NICE guideline recommendation of waiting for 2 days does not appear to be informed by research, and it is also unclear whether the 2-day timeframe is from the start of symptoms or from first consultation.^17^ The findings from the review presented here suggest this timeframe may be too short, with few participants likely to have improved within 2 days, although approximately a third will have improved by 7–10 days. The low rate of serious complications supports the practice of delayed prescribing to see whether symptoms self-resolve, before treatment is commenced, if required. These findings may assist clinicians to engage in collaborative decision making with their patients and discuss the possible course of the illness, with and without antibiotics, and to consider the benefits and harms of antibiotics. Information about the expected course of untreated UTI may also help patients to frame their expectations about recovery timeframes.

Certainty about the natural history of uncomplicated UTIs would be increased by additional primary research that, ideally, would involve rigorous studies with appropriate sample sizes, follow-up beyond the first few weeks of symptoms, use of standardised outcome measure descriptions and timing, and studies that include men. Further primary research is also needed for promising non-antibiotic treatments for UTIs, for example, non-steroidal anti-inflammatory drugs or increased oral fluids.^19^^,^^20^

## References

[1] Ellis AK, Verma S (2000). Quality of life in women with urinary tract infections: is benign disease a misnomer?. J Am Board Fam Pract.

[2] Falagas ME, Kotsantis IK, Vouloumanou EK, Rafailidis PI (2009). Antibiotics versus placebo in the treatment of women with uncomplicated cystitis: a meta-analysis of randomized controlled trials. J Infect.

[3] Bakhit M, Hoffmann T, Scott AM (2018). Resistance decay in individuals after antibiotic exposure in primary care: a systematic review and meta-analysis. BMC Med.

[4] Hoffmann TC, Del Mar C (2017). Clinicians’ expectations of the benefits and harms of treatments, screening, and tests: a systematic review. JAMA Intern Med.

[5] Coxeter PD, Del Mar C, Hoffmann TC (2017). Parents’ expectations and experiences of antibiotics for acute respiratory infections in primary care. Ann Fam Med.

[6] Altman DG (2001). Systematic reviews of evaluations of prognostic variables. BMJ.

[7] Brooks D, Garrett G, Hollihead R (1972). Sulphadimidine, co-trimoxazole, and a placebo in the management of symptomatic urinary tract infection in general practice. J R Coll Gen Pract.

[8] Christiaens TCM, De Meyere M, Verschraegen G (2002). Randomised controlled trial of nitrofurantoin versus placebo in the treatment of uncomplicated urinary tract infection in adult women. Br J Gen Pract.

[9] Ferry SA, Holm SE, Stenlund H (2004). The natural course of uncomplicated lower urinary tract infection in women illustrated by a randomized placebo controlled study. Scand J Infect Dis.

[10] Ferry SA, Holm SE, Stenlund H (2007). Clinical and bacteriological outcome of different doses and duration of pivmecillinam compared with placebo therapy of uncomplicated lower urinary tract infection in women: the LUTIW project. Scand J Prim Health Care.

[11] Continence Foundation of Australia Your guide to UTIs.

[12] O’Kane DB, Dave SK, Gore N (2016). Urinary alkalisation for symptomatic uncomplicated urinary tract infection in women. Cochrane Database Syst Rev.

[13] Asbach HW (1991). Single dose oral administration of cefixime 400mg in the treatment of acute uncomplicated cystitis and gonorrhoea. Drugs.

[14] Dubi J, Chappuis P, Darioli R (1982). [Treatment of urinary infection with a single dose of co-trimoxazole compared with a single dose of amoxicillin and a placebo]. Schweiz Med Wochenschr.

[15] Little P, Merriman R, Turner S (2010). Presentation, pattern, and natural course of severe symptoms, and role of antibiotics and antibiotic resistance among patients presenting with suspected uncomplicated urinary tract infection in primary care: observational study. BMJ.

[16] Gupta K, Hooton TM, Naber KG (2011). International clinical practice guidelines for the treatment of acute uncomplicated cystitis and pyelonephritis in women: a 2010 update by the Infectious Diseases Society of America and the European Society for Microbiology and Infectious Diseases. Clin Infect Dis.

[17] National Institute for Health and Care Excellence (2018). Urinary tract infection (lower): antimicrobial prescribing.

[18] Therapeutic Guidelines Limited Treatment of acute cystitis in adults.

[19] Kronenberg A, Bütikofer L, Odutayo A (2017). Symptomatic treatment of uncomplicated lower urinary tract infections in the ambulatory setting: randomised, double blind trial. BMJ.

[20] Scott AM, Clark J, Del Mar C, Glasziou P (2020). Increased fluid intake to prevent urinary tract infections: systematic review and meta-analysis. Br J Gen Pract.

